# Distinction without Separation. A Letter to the President of the College of Surgeons on the Present State of the Profession

**Published:** 1831

**Authors:** 


### XVIII.

Distinction without Separation. A Letter to the President of the College of Surgeons on the present State of the Profession. By Joseph Henry Green, F.R.S. &c. &c. Octavo, pp. 48. Sewed. May, 1831.

Those who are acquainted with Mr. Green—or who have heard his eloquent, scientific, and philosophic lectures at the College of Surgeons, will naturally be anxious to know the opinions of such a man on the present distracted state of the profession in this country. This, at least, was the case with ourselves, and therefore the instant we received the pamphlet, we read it through attentively. Mr. Green may be considered as a liberal reformer—but we fear his Bill will be considered *revolutionary* by the ultra-ar[illegible]ocracy of the profession; while it will appear not quite adequate to the wants or wishes of the democracy! Mr. Green is a profound reasoner, and his lucubrations are entitled to respectful consideration. His proposed plan of reform would, we think, be a decided improvement on the present system; and would very probably lead to improvements of a still higher character. As a distinguished ornament of that College whose cause he advocates, his proposals can hardly fail to make a strong impression on the existing rulers of the institution, though we can hardly hope that they will be disposed to adopt his suggestions to the full extent. But we shall now endeavour to lay a full account of this pamphlet before our readers.

With the following philosophic reflection the little work is opened.

"Professions, like States, seem destined to have their phases and mutations. They struggle from ignorance into light, rise through error and prejudice to their full meridian glory, decline then in the subdued splendour of forms that have no longer the inward life that produced them, and sink at last into the darkness of formulæ, dicta, and precedents, the *caput mortuum* of a creative intelligence. If we look to the professions of Divinity and Law, (to the Sciences I mean, as distinct from the professors,) I fear it cannot but be confessed that these sister stems, which entwining with Medicine, form the great trunk of the mind of the State, have ceased, in a great measure, to push forth their leaves and blossoms, and shew at present so few signs of life and living energy, that it might be doubted whether the sap continues to arise from the common root. Without attempting to disparage the useful labours of theologians and lawyers, for we are now speaking of the *Sciences* only of Theology and Law, not of the *men*—in the *literature* of these professions, we shall, I fear, look in vain for those marks of vigour which characterise the works of our elder divines and lawyers of that golden age of our national intellect, comprised in the reigns of Elizabeth, her immediate successors, and the Commonwealth: their teaching, preaching, writings, and works, were not the records of precedents, the amassment of mere facts, but breathe an intellectual life into all the knowledge of the age, into all its feelings, hopes and aspirations, and address themselves to the whole heart and mind of man."

Mr. Green then passes a well-merited eulogium on the medical profession for its scientific and literary attainments, its humanity, and the respectability as well as influence of its members throughout every ramification of society —through every region of the globe. But the eloquent author aptly observes that, if the medical profession is justified in claiming this rank, "it is bound to shew its credentials, and prove that its members have the desire and disposition, as far as their circumstances permit, of fulfilling and realizing the idea that animates or ought to animate their pursuits." This leads Mr. Green to a rapid retrospective glance at the origin of our medical institutions in this country—a retrospection, by the way, not over flattering to the beau-ideal character of the profession which the author had just drawn.

"If we look back into the annals of the medical profession in this country, we shall find in the charters granted to the College of Physicians, and to the Barber Surgeons, that the first attempt to its more effectual incorporation was not unpromising, and began with no inauspicious omen of future greatness. But it is no less true, that unfortunately at that time likewise were the Cadmean teeth sown, *mortalia semina dentes*, which were afterwards to start up in the armed forms of jealousy, rivalry, dissension, and separation, between the two important branches of physic and surgery. This might indeed have been prevented, had the College of Physicians acted up to their original charter; and had they continued to enforce its provisions, the right and duty of practising surgery being vested in them, the present College of Surgeons, or its humbler predecessor, the Corporation of Surgeons, would not probably have existed. And although it cannot be doubted that, sooner or later, the functions of the surgeon and physician, (at least in all large towns,) would have been exercised by different individuals, yet the well-educated surgeon would have been essentially a physician, fitted to be such by his education, and by virtue of his attainments admitted as a member or fellow of the College of Physicians; and the difference in the two classes of physicians and surgeons would have been only *practical*, whilst the absurdity of a division, where it is impossible to draw a boundary line, would have been obviated: there would have been, I say, *distinction* without *separation*. There would then have been a scheme laid of ordered unity, for which the elements must have their rank assigned by a principle of co- and sub-ordination, in default of which the elements become hostile opponents. But, unfortunately, whatever the cause may have been, instead of this distinction, which not only permitted, but might have secured and perfected, a union, the College of Physicians resorted to an exclusive division from the surgeons; and we find them taking no other interest in the proceedings of the latter, than by instituting law-processes against them, or the more summary mode of vindicating their supposed rights, by committing them to Newgate. And thus the circumstance of the distinction of function with a common object, instead of acting to fraternize and cement, became the deadly poison of jealousy and hatred."

But the surgeons, though disgraced by their fraternization with the Barbers, began gradually to rise into importance, and ultimately effected a separation from their ignoble brethren of the razor, and the establishment of a corporation for themselves. Here we have to deplore a division which weakened the profession, and seemed to erect an impassable boundary between the two great classes of medical practitioners. But this was not all. A third division or sect was soon to be created more numerous, and perhaps more powerful than both the other two—the General Practitioners. Originally only the dispensers of the physician's prescriptions, they were afterwards entrusted to administer—and ultimately to prescribe medicines. Native talent and the diffusion of knowlege have now rendered this class physicians in ordinary to the community at large.

"Their surgical functions brought them of course into connexion with the surgeons, as the surgeons in many instances arose, from very obvious causes, out of the class of general practitioners. But although thus every way connected in interest, and united in a common object with the higher branches of the profession, we find, as the advance of knowledge required a closer examination into the qualifications of the general practitioner, that in an inauspicious moment, the regulation of this department of the profession was entrusted, by Act of Parliament, to the trading company of Apothecaries, and thus a new and independent interest created in the profession. And although the society of apothecaries have, with exemplary zeal, directed their efforts to promote the better education and respectability of their members; yet it would be difficult to assign any sufficient reason for their selection to so important a trust; and we have to regret that it presents another instance of separation, where distinction without division might have been easy, and has become, like the separation of surgery from physic, another cause of disunion and weakness in the medical profession.

Thus, throughout the medical profession, in which there is essentially a community of interests and objects, instead of promoting and cementing a union of its different departments, which would have given strength to the higher, and an increase of respectability to the subordinate, their interests have been rendered independent, and conflicting, to the great danger of its stability, and with the obvious tendency of crippling its energies and of lessening its sphere of utility."

This is a melancholy, but true picture; and the learned author despairs of a remedy—"the separation of the different departments of the profession being a thing *jam consummatum*, and, for the present at least, irremediable."

Mr. Green then proceeds to investigate the origin, the intentions, and the conduct of the College of Surgeons—expressing an earnest and honest desire "to discover the foundation (if any) for those bitter complaints, and loudly-trumpetted-forth grievances, which, full of sound and fury, threaten to shake to its foundations the College of Surgeons." Mr. Green proceeds to a very recondite, and to us not always intelligible discussion into the nature or colleges, societies, or institutes, for the protection or promotion of science or the fine arts, as distinguished from corporations, guilds, and companies for the regulation of commerce, or the maintenance of rights and privileges. In the latter class of institutions our author argues that it is right and proper that a certain number out of the whole class should be elected to watch over the common interests of all, in which process, the electors can find easy and sufficient criteria in the wealth, extensive dealing, and fair character of the candidate or nominee. From the following extract, it will be seen that Mr. Green takes a very different view of scientific corporations.

"Now, if, on the other hand, we look into the history of the different great learned or scientific colleges, academies, and institutes of civilised Europe; or if we consider the ends and purposes of their institution; we shall find that the paramount object has been to create a class not already existing, or to call forth a class existing only under the scum of such imperfections and deformations as necessarily intercepted every form of excellence that might be contained virtually therein. The purpose, I repeat, of the illustrious founders, has been to take advantage of the fortunate accidents of genius, knowledge, and attainments, which the particular age and country had presented, and so to combine these, as that they should work productively as well as influentially on the mass successively subjected to their influence, so as, in the greatest possible degree, to assimilate it to themselves. They were the ferment that was to work in the production of a given body, and not merely to be choice specimens of products already existing. The colleges of learning and science may exercise various functions, and fulfil sundry purposes, which belong to the corporations of common life; but this is their peculiar character, this is that by which they have and can alone worthily retain the name of a learned or liberal incorporation—that their characteristic object is prospective, the promotion, the advancement of the science or art, in distinction from, though, thank God, in necessary union with, the interests of scientific men, as individuals. Their characteristic mode of action is to work by descent; they are to be the suns of the system to which they belong, and not mere mirrors, reflecting only the light that had been previously bestowed; and their characteristic form, from the very beginning, is by *appointment* —appointment by a higher, in contradistinction from *election* by a supposed lower, or equal."

"Appointed thus from the highest authority, and "exercising an influence, which evermore works *descensively* till, as the product of its own subliming and assimilative action, a correspondent ascension gradually takes place, a college thus framed perfects itself into a circle, ever working *from above*, yet ever returning on itself." These are sublime images of beau-idealism in surgery, which we love to dwell on; but unfortunately we cannot shut our eyes to the realities of life around us! When we contemplate these "suns of the system to which they belong," radiating their beams of knowledge on the innumerable distant orbs of the same system in Lincoln's-Inn-fields—when we see these distant orbs "ever working from above"—and so prone to amalgamate with the "suns" of the system, that it requires talismanic wands to keep them from rushing into terrific collision—we cannot but think that the ingenious author of the brochure before us, has drawn a little upon his own fertile imagination — and portrayed that which *ought* to be, rather than that which *is*.

The able writer goes on to state that the great purpose or object of a learned body is to confer honour, to delegate authority — to give assurance to the community of competent skill—to exclude from the ranks of the profession every unworthy member. That these praise-worthy objects are all accomplished by the College of Surgeons, seems to be doubted, even by the College's own professed advocate.

"How far the college has answered the purposes for which it was intended? whether it has produced all the advantages that might have been expected? or again, whether any improvements might be suggested for its more efficient administration?—these are questions which are now to occupy us, and the answers to which are to form the results of our inquiry. That it has done much good, I cannot doubt, in producing a better educated and more efficient body of surgeons, in rendering them more anxious for professional character, and in raising them in the public estimation. But whilst the college possesses no power of preventing ignorant and unqualified persons from practising surgery; whilst men of bad character are permitted to enjoy the privilege of being associated with men of honour and respectability; and whilst advertising quacks are suffered to stand on the list of its members, it may be doubted whether its influence is such as might be wished; nor am I prepared to say, that the examination of candidates for admission, and the lectures illustrative of the museum, in point of extent, are such as may be deemed worthy of the advanced state of science."

Mr. Green denies that these defects in the institution were the causes of dissatisfaction upon recent occasions—and he maintains that the said dissatisfaction is limited to a small fraction, or faction of the members at large.

"On the contrary, it is notorious that the loud and the turbulent, as in most cases, form but a small, and that the least respectable part of our community; and that not only has there been no complaint nor remonstrance by any number, however small, of respectable members, in respect of any mal-practice or mal-administration of the college, but it is evident from the still swelling list of members, that the candidates for general practice are anxious to obtain the college diploma, and consider it an honour and a proof of their respectability to belong to the college. *I will not, however, attempt to conceal that discontent and dissatisfaction exist, and that many of the members of the college who have not given a public expression of their feelings, perhaps from fear of being identified with persons whose motives and objects they disapprove, still sufficiently show that they are by no means cordial friends of the establishment, and evince, in various ways, their dislike and jealousy of college influence.* I believe it will be difficult to discover the nature, and trace the causes of this more or less prevalent feeling. The general practitioners feel themselves,—in consequence of their ineligibility to places of influence, and from having no voice in the affairs of the college,—under a perpetual ban of humiliating disparity, in comparison with those who practice surgery exclusively; and I apprehend that the discontented generally would, (if they could divest themselves of the sense of propriety, the absence of which characterizes the turbulent few,) *adopt the same language as the latter have done, and demand for themselves eligibility to the council, the power of electing members to the same, and the control of the application of the funds.*"

The author would gladly believe that this feeling is *not* general among the surgeon-apothecaries, since he could not ascribe it to any desire of increasing the respectability of the profession, which ought to be the aim of its members. He therefore attributes this feeling to a "spirit of restless ambition, and the delusive lust of individual power." The sum and substance (he maintains) of the defects complained of in the present constitution of the college, are the irresponsibility of the council—its powers of self-election—and "the exclusion of the surgeon-apothecaries from all control of the funds, and from places of trust, as well as from the elective franchise." In respect to the *irresponsibility* of the council, the author meets the objection by asking if the council have abused their trust, or rather their absolute power? We are rather surprised to see so sensible a writer as Mr. Green make use of so weak an argument. Is unlimited power to be confided to a monarch because he has never abused power or acted the tyrant? History surely has not been consulted in vain by Mr. Green. After vindicating the conduct of the council (and we are far from accusing them of any misconduct) the author appears to feel the weakness of the argument, and endeavours to shew, by a somewhat subtle and forensic train of reasoning, that the council is *not* irresponsible. The author shall speak for himself.

"But is the council, according to the previous assumption, really irresponsible?—So far from it that, according to the laws of England, the King, who is the founder of all corporations, is constituted by law the visiter of the same, and he exercises this jurisdiction in the Court of King's Bench, where all misbehaviours of corporations are inquired into and redressed, and which, upon a proper complaint and application, can prevent and punish any injustice of which they may be found guilty. Thus, then, for the due administration of the affairs of the surgical profession, there do not appear to be any sufficient grounds for removing it from the hands in which it is placed."

That our Patriot King is ever ready to attend to and redress the grievances of his subjects, we well know; but that His Majesty should take upon himself, through the medium of his "Court of King's Bench," to rectify the grievances of the members of the College of Surgeons, is rather too much for common sense! This indeed is the weakest part of the pamphlet, and much are we astonished that Mr. Green should so openly expose the weakness of his cause. Mr. Green's arguments against *universal suffrage* in the election of the council and other offices in the college, are better supported; and as this is one of the most important of all the questions discussed, we shall again allow the author to speak in propria persona.

"And I proceed, then, to the remaining question, that of the power of nomination and appointment, vested in the council, and the consequent exclusion of surgeon-apothecaries from eligibility thereto, and from any elective franchise. And although this question might be fairly dismissed, by referring to the distinction already established between scientific institutions and trading corporations, in drawing which it has been shewn, that the former can be in no other way renewed and perpetuated than by taking up, receiving, and appointing those, whose qualifications and attainments fit them to carry on the original purpose of the institution, and that the very objects and intentions of such learned bodies would be frustrated by popular elections; yet if, admitting even the principle of election, we can shew that the admission of surgeon-apothecaries to the same privileges as those members of the College who practice surgery exclusively, would be neither just nor desirable, nor even practicable; and that this must be granted by any reasonable man, or even by any general practitioner not wholly blinded by his passions,—then I presume it will be admitted, that the question respecting such a change in the constitution of the College, may be suffered to fall to the ground. Now it is plain, and so acknowledged by the public, that the surgeon-apothecary derives a large share of his respectability and of his general professional character, and receives the confidence of his patients in consequence of his being associated with, and having received the diploma of the college; and that it not only is so, but that the general practitioner feels it himself, and is proportionally anxious to make it known that his qualifications for practising surgery have been examined into and approved by the college. It would be wonderful if it were otherwise, for the testimonial of his ability is guaranteed by the signatures of those whose names are well known to the public, as men whose knowledge and judgment are the best warrants and authorities for the truth of the certificate. Substitute for the names of men eminent in character those who, however much and deservedly prized within a narrow circle, are not known to the public at large, and the diploma will become little better than waste paper, or at any rate comparatively insignificant and worthless. And surely if the end aimed at in the selection of the council be that of exalting the dignity of the profession, that end will be best attained by selecting and raising those who are most distinguished and eminent as surgeons; whereas by putting on an equality those who practice surgery exclusively, and those who make surgery a subsidiary qualification, and making the latter eligible to all plans (places) of honour and trust, the object of the institution of the college, which is that of promoting the science of surgery, and not the interests of the practitioners, will be defeated, and the college would in all probability dwindle into a trading corporation: *not to mention that I fear that, as the general practitioners are in an overwhelming proportion to the surgeons, it might not improbably happen, that the latter would be excluded altogether.* And I believe we may state it as a general truth, that by a system of equalization, the higher being brought to a level with the lower, this process of levelling would infallibly vulgarize the profession; whilst on the other hand, the more you honour that which is distinguished, the more that which is lower will be raised, partake of the honour, and derive an increase of its estimation with the public.

"I trust, however, that my meaning will not be misunderstood, as if I meant to undervalue the attainments and talents of general practitioners, since I am only speaking of their share in the profession of surgery as being necessarily lower, because forming really a subordinate part of their practice, in comparison with those who practice surgery exclusively. I cheerfully admit that the general practitioners number amongst them men most estimable in talent and character, yet they themselves will allow that the avocations and pursuits of the great body of their members, do not exactly fit them for the guardians of professional honour, and the promoters of science; and without wishing even to insinuate that those who devote themselves exclusively to surgery, are men of more integrity or more talent, yet I cannot but believe that their habits as teachers, as surgeons of hospitals, their residence in London, and intercourse with those most influential in rank and talent, render it more likely that they should take enlarged views; that they should pursue professional studies with a view to the cultivation of science, and thus liberalising the profession, free it from the petty interests of a trade; that they should be awake to the wants of the profession, and supply them without jealousy or partiality; in short, that both their habits and their local advantages best fit them for constituting the governing council of the surgical department of the profession. It does appear, therefore, that it is not without justice, nor without consulting their own best interests, as professional men, that surgeon-apothecaries are, in the *present* state of the profession, excluded from forming a part of the executive administration of the affairs of the college."

But admitting that the General Practitioners should be considered ineligible for admission into the council, it may still be argued that they ought to have a voice in the election of the pure surgeons to that high office. Allowing this elective franchise to be conferred on the members at large, Mr. Green asks who are those who would virtually exercise it? He answers—"the London Practitioners, since it is impossible that the country practitioners, the surgeons of the army and navy, or those resident in our colonies, could attend the election." He contends then that the elective franchise, designed to give influence to the majority, would, in fact, "give power to the few at the expense of the many." There is certainly considerable force in this objection to universal suffrage in surgery.

"But in thus adverting to the practitioners in the country, I cannot pass over the fact, that there is a very important distinction to be made between many, or most of these, and the London practitioners. In the country, necessarily, a great deal more of surgical practice devolves upon the general practitioner than in London; and many being surgeons of county hospitals and infirmaries, and residing in large towns, like Manchester, Leeds, Birmingham, Liverpool, (names which bring to my recollection men most eminent in practice, and sedulous cultivators of their profession as a science,) there would be found amongst them most desirable acquisitions, and men who would form bright ornaments to the college council: were it not obvious, that to the availing ourselves of their services, their residence in London is an indispensable condition. On the other hand, the London general practitioners, seldom called on in pure surgical cases, or acting only in a subordinate capacity, are therefore, of the whole body, those least fitted to direct and control the interests of surgical practice and science."

But Mr. Green is not so Utopian as to expect that the above and many other arguments against popular elections and universal suffrage, will give any thing like general satisfaction to "those who desire a larger share of influence and power;"—and so far he would be disposed to go with them—"not perhaps in agreeing with them as to the remedy, but in deploring as an evil, in the present constitution of the College, the *want of sympathy* or communion between the members at large, and the governing body." This is, unquestionably, an evil of tremendous magnitude; for it will constantly bring, not only into public discussions, but into private life, *unceasing* enmity and hostility subversive of the respectability of the profession! Under these impressions the talented advocate of the College holds it to be a legitimate object of inquiry whether there are "any means of producing a greater confidence in the College, a closer union of its members, and a probable extension of its influence and benefits?" Mr. Green thinks it may perhaps be possible so to modify the charter, as to "satisfy the excluded, and thereby strengthen the College without interfering with the principle of its foundation." We are now prepared for the plan proposed by Mr. Green.

"1. That the government of the College should be vested in a *President*, a *Supreme Council*, and a *General Council*.

2. That the Supreme Council should consist of the President and twenty members, who should have the entire management of the affairs of the College, and the conducting of examinations.

3. That the members of the Supreme Council should appoint its own members from the General Council, and consist only of those who do not practise midwifery, nor dispense medicines.

4. That the General Council should consist of the members of the Supreme Council, and of forty additional members, twenty of whom should be under the obligation not to practise midwifery nor dispense medicines, and the remaining twenty of general practitioners—making the total number of the General Council *sixty-one*.

5. That the General Council should appoint its own members.

6. That the General Council should choose auditors of the accounts, and might suggest to the Supreme Council at their meetings any measures for the benefit of the profession. And further, that all public acts of the Supreme Council should be communicated to them.

7. That the eligibility of that class of members of the General Council, under the obligation of not practising midwifery, nor dispensing medicines, should be further determined by proofs of a longer course of study, and of superior capability, evinced by severer examinations. 1. On entering the profession, they should produce certificates at the College of having been instructed and undergone examinations in *Latin, Greek, Mathematics*, and *Logic*. 2. That they should have devoted at least *five years* to the study of their profession, and produce certificates of having attended lectures on anatomy, physiology, chemistry, materia medica, botany, practice of medicine, medical jurisprudence, comparative anatomy, midwifery, and that during that time they have attended a public hospital. 3. That they undergo three distinct examinations; the first on anatomy and physiology, the second on pathology and therapeutics, and the third on surgery: and that they write *a thesis* on a given subject, in a closed chamber, without the aid of books.

8. That there should be a class of Honorary Members of the General Council, men of distinguished merit in provincial towns, the army, navy, or colonies.

9. That general practitioners who have given up the practice of midwifery and the dispensing of medicines, should be eligible to the first class of the General Council.

10. That teachers of anatomy and surgery should not only have undergone the examinations of the first class, but should have given *public proofs* of their capability to teach by delivering a lecture, the preparation for which should not occupy more than twenty minutes.

11. That effectual means should be taken of enforcing the duties of masters to their apprentices or articled students, by a prescribed and definite course of instruction.

12. In the provisions of a new Charter, it should be imperative that no one should be allowed to practice surgery who was not a member of the College. Since without this check upon ignorance and empiricism, it is impossible that the College can exercise one of its most important functions —that of protecting the public from the arts and practices of dishonest, unskilful, and incompetent pretenders.

13. And lastly, that the Charter should distinctly define, express, and declare, the power of expelling all those who, by dishonourable practices, have rendered themselves unworthy the character of members of a liberal profession, whether it be by the use of secret remedies, by advertizing, by partnerships in trading concerns, by calumnious reports of their professional brethren, breaches of professional confidence, or whatever else may be considered derogatory to a professional character."

We think that no unbiassed member of the medical profession can read the foregoing proposal, without conscientiously admitting that it would be a measure of incalculable benefit to the surgical profession, as compared with the present system. Much as we are disposed to raise our voice against all arbitrary power, we nevertheless most heartily approve of the concluding, or 13th article—viewing it as a kind of court of honour for punishing those numberless dishonorable and disreputable practices which could not possibly come under the cognizance of a court of law. Let us just apply the jurisdiction of such a court (which should, of course, embrace the whole 61 members) to the present moment. If the College of Physicians possessed the power, or had the inclination to exercise such a wholesome control over its members, would the vile epistle in favour of St. John Long, and vituperative of all that is respectable in the profession, ever have emanated from Ely Place? Long experience and no inconsiderable communication with all ranks of the profession, have impressed us deeply with the necessity for some COURT of HONOUR, of the above description. We are firmly convinced that it would be of more utility than all the proposals contained in the twelve preceding articles of reform put together. But the best part of the whole pamphlet is yet to be presented to our readers.

"In addition, however, to all this, I would have the college, with a bolder zeal, promote science from its ample resources, especially by extended courses of lectures on anatomy, physiology, and pathology; on comparative anatomy, and on surgery—lectures, which might do justice to Mr. Hunter's museum, and prove worthy of the college, and of the present advanced state of science.

"Nor should I think the plan complete without the publication of *transactions*: and I would suggest that these should not only consist of voluntary contributions, but that each member of the general council should, on his admission, be obliged to furnish a paper; that they should moreover contain digested *reports* from the hospitals, both in town and in the country; and that a foreign secretary should be appointed who might correspond with the hospitals and scientific institutions for the promotion of surgery on the continent, and in America, and maintain a communication with medical men throughout the globe.

"If the college shall do all this, I think it cannot fail in healing all differences with its members, or if it have opponents, in shaming them into silence; and in raising itself in the public estimation, it will deserve the gratitude of the public and of the profession. And I do not doubt that as, in order to carry these beneficent objects into effect, a modification of the charter would be necessary, that all the respectable members, in other words, the majority and great body of the profession, would cheerfully join with the college in *petitioning* for this enlarged charter, which would secure the interests of science, the progressive improvement of the profession, and hope for all candidates for its privileges and honours.

"But if such a petition were presented to me as a minister of state, appointed to consider the provisions of such a charter, I should pause before I granted it. For I should be led to reflect on the state of the whole medical profession, and considering its vital importance to the state, its objects and purposes, I should come to the conclusion, that however desirable it may be for its practical administration, that its departments should be distinguished, yet that from the unity of its character and purposes, they could not be divided. Instead, therefore, of any partial alteration or regulation, I should advise that ONE faculty of medicine be constituted, with such powers and administrative regulations as would render it efficient in promoting the science, and controlling the practice of medicine in all its branches, as a great interest of the state. Of this faculty, the colleges of physicians and surgeons, as representing the great leading distinctions of the profession, would naturally form the co-ordinates. In order to the admission of candidates to either, it might be required that they should have passed through the same course of study, which should be upon the most extended plan of a liberal and professional education, and that the examinations for ascertaining their proficiency, should be conducted by both; and that then from the candidate expressing his wish to enrol himself in either, as intending to devote himself practically to one or other branch pre-eminently, whether medicine or surgery, such additional proofs of competency might be required, as might shew that he was entitled to the desired privilege. And thus the practical distinction between medicine and surgery would be acknowledged, whilst their scientific unity would be preserved.

"Out of both would then naturally arise a third department, partaking of the character of each,—that of midwifery. This might have its separate board or institute, and the candidates for admission having the same basis of general education, would follow a similar rule for the enrolment of its members, by requiring a special skill and knowledge in this department of the profession.

"Next, as conjoining the functions of all three, the class of general practitioners would find its place: their institute forming a department of the faculty, which would in like manner regulate the admission of candidates, their education and qualifications, and watch over the affairs of their particular branch of the profession.

"Lastly, from the colleges or institutes of medicine, surgery, midwifery, and general practice, might be formed a medical convocation, for the purpose of deliberating on all matters relating to the profession at large. And thus a body would be constituted in the service of the state, with whom the government might consult, and to whom the country would look for advice and assistance in all matters appertaining to the health of the community, and to whom all questions relating to epidemics, laws of quarantine, the health of the army and navy, the building of hospitals and prisons, punishments, drainage, sewers, nuisances,—in fine, all questions of medical jurisprudence and police might be referred. And to a faculty of medicine so constituted, might be entrusted the government and supervision of the practical departments of the profession, and that not only should none practice medicine, surgery, or midwifery, without their sanction, but that all keepers of houses of reception for lunatics, all druggists and chemists, dentists, cuppers, should be obliged to have their licence for their several callings. And if the government would render the benefit complete and national, they would root up the detestable upas-tree of quack and patent medicines. And thus, Sir, we might at length see a profession flourishing in this country, the motto of which would be DISTINCTION *without* SEPARATION."

It will be evident that this plan does not differ very essentially from that which has been recently proposed by other medical reformers, under the title of "THE LONDON COLLEGE of MEDICINE." As being much more likely to succeed, if consented to, and assisted by the "POWERS THAT BE," we would strenuously advise a general movement in the profession to promote the measure. If there be any life or spirit in the ranks of medical society, we call upon them to unite, on this important occasion, and by a wise, temperate, but firm petition to the Colleges of Physicians and Surgeons—or even to the Legislature, endeavour to bring about such a union, such a concours, as that proposed by Mr. Green. No man is better qualified to preside over an association of the profession for this purpose, than the distinguished surgeon last-mentioned, and, if they do not call a meeting, and invite him to the chair, they deserve to remain in disunion, disgrace, and derision to the end of the chapter. A committee of physicians, surgeons, and general practitioners should be instantly formed to deliberate and act on this all-important measure. Now is the time! Let no petty jealousies distract our councils. Let the reformers who have taken the field, unite their efforts with those of more moderate, but more feasible principles, and the victory will be certain. With such a man as Mr. Green for president, the brightest ornaments of the profession, in town and country, will hasten to offer their support, and contribute, in every possible way, to the paramount object of reform. We repeat it, that the measure only wants a starting-point to insure it success. All party-feeling should be detruded from a convocation where the universal good of the profession is the end in view. We shall probably return to the subject before this number is closed.

